# The prognostic value of tumor-infiltrating T lymphocytes in ovarian cancer

**DOI:** 10.18632/oncotarget.14919

**Published:** 2017-01-31

**Authors:** Jun Li, Jieyu Wang, Ruifang Chen, Yang Bai, Xin Lu

**Affiliations:** ^1^ Department of Gynecology, Obstetrics and Gynecology Hospital, Fudan University, Shanghai 200011, China; ^2^ Shanghai Key Laboratory of Female Reproductive Endocrine Related Diseases, Shanghai 200011, China

**Keywords:** ovarian cancer, tumor-infiltrating lymphocytes, prognosis, survival, meta-analysis

## Abstract

The prognostic value of tumor-infiltrating lymphocytes (TILs) in ovarian cancer is still in controversial. This study is aimed to assess the impact of different TIL subsets on the progression free survival (PFS)/disease free survival (DSS) and overall survival (OS)/disease specific survival (DSS) in ovarian cancer. A comprehensive literature search in PubMed, ISI Web of Science, and Medline was performed to identify relevant studies evaluating the prognostic value of TILs in ovarian cancer. Reviews of each study were conducted and data were extracted. The main outcomes analyzed were PFS/DFS and OS/DSS. A total of 21 eligible studies enrolling 2903 ovarian cancer patients were included for the meta-analysis. The overall analysis revealed that intraepithelial CD3^+^ and CD8^+^ TILs were strongly associated with improved PFS/DFS (HR=0.53, for CD3^+^ TILs; and HR=0.50, for CD8^+^ TILs). Intraepithelial CD8^+^/Foxp3^+^ ratios appeared to be associated with improved PFS, though without reaching statistical significance (HR=0.73). Moreover, intraepithelial CD3^+^, CD8^+^, and CD103^+^ TILs were clearly associated with increased OS/DSS (HR=0.50, for CD3^+^ TILs; HR=0.62, for CD8^+^ TILs; HR=0.54, for CD103^+^ TILs). However, intraepithelial FoxP3^+^ TILs, CD8^+^/FoxP3^+^ ratios, CD8^+^/CD4^+^ ratios, and stromal TILs had no impact on the OS/DSS (HR=0.98, for FoxP3+ TILs; HR=0.69, for CD8^+^/FoxP3^+^ ratios; HR=0.48, for CD8^+^/CD4^+^ ratios; HR=0.82, for stromal TILs). In conclusion, the present meta-analysis supports the hypothesis that intraepithelial TILs are predictive biomarkers for the prognosis of ovarian cancer patients. Future randomized studies are needed to verify these observations.

## INTRODUCTION

Epithelial ovarian cancer accounts more deaths annually than other gynecologic cancers [1]. Although prognosis of early-stage disease is favorable with a 5-year survival rate approaching 90%, the majority of the patients are not diagnosed until advanced stages. With cytoreductive surgery and platinum based chemotherapy, more than half of such patients will achieve remission, however, most cases will succumb to platinum resistance and disease progression [2]. Until now, no effective biomarkers have been identified that can reliably predict the prognosis of ovarian cancer. Thus, there is an urgent need to search for more informative diagnostic and prognostic factors for such patients.

Increasing evidence indicates that ovarian cancer is an immunogenic disease that can be recognized by the host immune system [3]. The interplay between the immune system and cancer cells is critical for tumor progression. Thus, in recent years much work has been entered into the detection and characterization of tumor infiltrating lymphocytes (TILs) in ovarian cancer [4, 5]. TILs are a type of white blood cells detectable in the tumor islet and stroma that recognizes tumor cells to cause immune response. The first report on the survival benefit of TILs in ovarian cancer was attributed to Ma in 1991 [6]. As then, many attempts have been made to document the prognostic value of TILs in ovarian cancer [7]. Zhang et al. [3] performed analysis on 186 snap-frozen specimens from advanced stage ovarian cancer and found that the presence of intratumoral CD3+ TILs were indicative of improved survival. However, a study by Sato et al. [8] failed to document the survival benefit of CD3+ TILs in ovarian cancer. By contrast, Sato et al. [8] demonstrated that intraepithelial CD8+ TILs were the only subtype associated with favorable prognosis in ovarian cancer. The discrepancy in results suggests that the prognostic significance of TILs in ovarian cancer remains controversial.

With the aim to gain a better insight into the prognostic value of TILs in patients with ovarian cancer, we performed a meta-analysis of published literature on this topic. In particular, we evaluated the effects of TILs status on the survival in ovarian cancer patients.

## RESULTS

### Characteristics of identified studies

**Figure 1:**
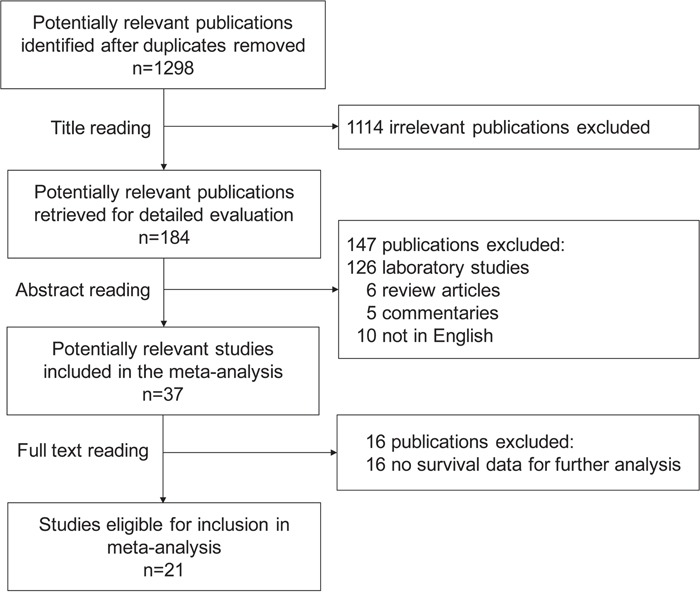
Flow chart of the search strategy used for selection of eligible studies

One thousand two hundred ninety-eight publications were identified by the primary computerized literature search. Of these, 1262 studies were excluded because they were either laboratory studies, review articles, commentaries, written in non-English, or irrelevant to the present study. Thirty-seven records were further reviewed in detail. Fifteen publications were further excluded because of no survival data. Finally, 21 studies were identified as eligible for inclusion in the meta-analysis (Figure 1). The included 21 studies encompassed 2903 ovarian cancer patients [3, 8–27]. The main characteristics of the included studies are shown in Table 1.

**Table 1:** Characteristics of included studies

First author of study, y	Country	No.of patients	Tumor stage	Grade	Histologic subtype	Specimen processing	TILs Phenotype	Location	Cut-off value	Outcomes	HR estimation
Zhang, 2003	Italy	174	III, IV	Mixed	Mixed	Cryosections	CD3	Intratumoral	Any cells/HPF	PFS, OS	Data extrapolated
Sato, 2005	USA	117	Mixed(III-IV, 88%)	Mixed(G2-G3,93%)	Mixed(Serous, 78%)	Paraffin-embedded	CD3,CD4,CD8,FoxP3	Intraepithelial, Stromal	Upper two tertiles (>3.3 cells/20xHPF)	OS	Reported in text
Raspollini 2005	Italy	95	III	High	Serous	Paraffin-embedded	CD3	Intratumoral	≥5 cells/HPF	DFS, OS	Reported in text
Hamanishi,2007	Japan	70	Mixed(III-IV, 55.7%)	NR	Mixed(Serous, 40%)	Paraffin-embedded	CD8	Intraepithelial, Stromal	≥5 cells/ 0.0625mm^2^	PFS, OS	Reported in text
Callahan, 2008	USA	184	III, IV	High	Serous	Paraffin-embedded	CD8	Intraepithelial	Top quartile	OS	Reported in text
Tomsova, 2008	Czech Republic	116	Mixed(III-IV, 74%)	Mixed(G2-G3,87%)	Mixed(Serous, 47%)	Paraffin-embedded	CD3	Intraepithelial, Stromal	>125cells/ mm^2^	OS	Reported in text
Han, 2008	USA	150	Mixed(III-IV, 91.3%)	Mixed(G2-G3,92%)	Mixed(Serous, 78.7%)	Paraffin-embedded	CD3/CD8	Intratumoral, Peritumoral	Any cells/HPF	OS	Reported in text
Leffers,2009	Netherlands	306	Mixed(III-IV, 69.6%)	Mixed(G2-G3,70.2%)	Mixed(Serous, 55.9%)	TMA	CD8, FoxP3	Intratumoral	Upper two tertiles as number of cells/mm^2^	DSS	Reported in text
Clarke, 2009	Canada	500	Mixed(III,16.8%)	Mixed(G2-G3,78.9%)	Mixed(Serous,42.4%)	TMA	CD3,CD8	Intraepithelial	Any cells/two 0.6mm cores	DSS, PFS, OS	Reported in text
Stumpf 2009	Germany	100	III	Mixed(G2-G3,97%)	Serous	TMA	CD8	Intraepithelial	Any cells/HPF	DFS/OS	Reported in text
Adams 2009	USA	134	III, IV	Mixed(G2-G3,100%)	Mixed(Serous,94%)	Cryosections	CD3,CD8, FoxP3	Intraepithelial	CD3(Any cells/HPF); CD8,FoxP3(10cells/HPF)	OS	Data extrapolated
Vermeij 2011	Netherlands	270	Mixed(III-IV, 65.4%)	Mixed(high,49.2%)	Mixed(Serous,59.8%)	TMA	CD8, FoxP3	Intraepithelial	Upper two tertiles as number of cells/mm^2^	PFS, DSS	Reported in text
Bachmayr-Heyda, 2013	Austria	203	Mixed(III-IV, 95.6%)	Mixed(G2-G3,96.1%)	Mixed(Serous,88.2%)	TMA	CD8	Intraepithelial	>median	PFS, OS	Reported in text
Mhawech-Fauceglia 2013	USA	73	Mixed(III-IV, 66%)	Mixed(G2-G3,93%)	Mixed(Serous,60.3%)	Paraffin-embedded	CD3, CD8, CD25, FoxP3	Intraepithelial	Any cells/HPF	OS	Reported in text
Webb 2014	Canada	497	Mixed(III,16.9%)	Mixed(G2-G3,78.9%)	Mixed(Serous,42.3%)	TMA	CD103	Independent of location	>5cells/0.6mm cores	DSS	Reported in text
DeLeeuw 2015	Canada	187	Mixed(III,34%)	Mixed(G2-G3,100%)	Serous	TMA	CD4,CD8,CD25, FoxP3	Intraepithelial	Any cells/HPF	PFS,DSS	Data extrapolated
Webb 2015	Canada	489	Mixed(III, 17%)	Mixed(G2-G3,78.7%)	Mixed(Serous,42.1%)	TMA	PD1,CD3,CD8,CD25,FoxP3	Independent of location	Any cells/0.6mm cores	DSS	Reported in text
Knutson 2015	USA	348	Mixed(III-IV, 86.2%)	Mixed(high,97%)	Mixed(Serous,81.4%)	Cryosections	CD4,CD8,CD25, FoxP3	Intraepithelial	Any cells/HPF	OS	
Bosmuller 2016	Germany	138	Mixed(III-IV, 85%)	High	Serous	TMA	CD3,CD8,CD103	Intraepithelial	CD3:≥7 cells/HPF; CD8:≥0.5cells/HPF;CD103:≥1cells/HPF	OS	Data extrapolated
Strickland 2016	USA	53	NR	High	Serous	Paraffin-embedded	CD3	Intraepithelial	≥35 cells/HPF	OS	Reported in text
Darb-Esfahani 2016	Germany	215	Mixed(III-IV, 86.6%)	High	Serous	TMA	CD3, PD-1, PD-L1	Intratumoral	CD3:>65cells/mm^2^; PD-1:>11cells/mm^2^;PD-L1:>20cell/mm^2^	PFS	Reported in text

### Intraepithelial TIL effects on survival in ovarian cancer

### CD3^+^ T lymphocytes

**Figure 2:**
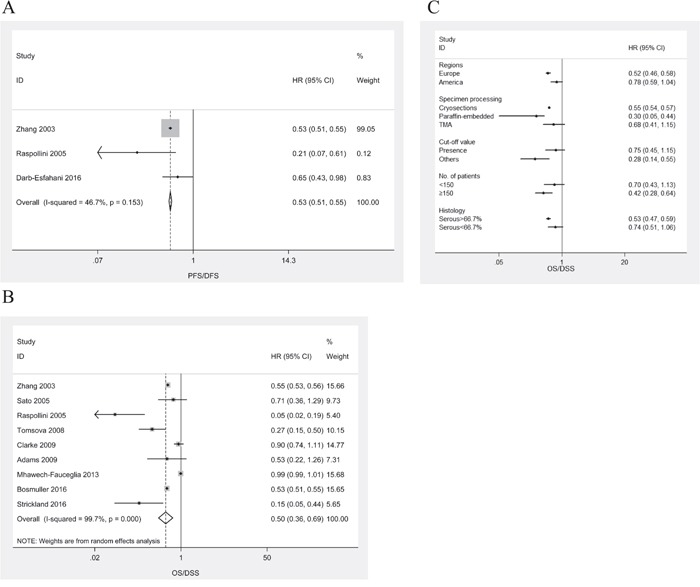
Meta-analysis of the HR for PFS/DFS and OS/DSS for ovarian cancer patients depending on intraepithelial CD3+ TILs status **A**. PFS/DFS for ovarian cancer patients, fixed effects model. **B**. OS for ovarian cancer patients, random effects model. **C**. Subgroup analysis stratified by various clinical variables.

HRs for PFS/DFS were available in 3 studies [3, 9, 27]. The estimated pooled HR for all studies suggested a significantly decreased risk of disease progression in patients with intraepithelial CD3^+^ TILs (Figure 2A; HR, 0.53; 95%CI, 0.51-0.55; P_HR_<0.001; I^2^=46.7%; fixed effects model). There was no publication bias (P_Begg_=1.00, P_Egger_=0.81). One-way sensitivity analysis confirmed the stability of our results (Supplementary Figure 1A)

HRs for OS/DSS were available in 9 studies [3, 8, 9, 12, 15, 17, 19, 25, 26]. The estimated pooled HR for all studies suggested a significantly decreased risk of death in patients with intraepithelial CD3^+^ TILs (Figure 2B; HR, 0.50; 95%CI, 0.36-0.69; P_HR_<0.001; I^2^=99.7%; random effects model). There was no publication bias (P_Begg_=0.75, P_Egger_=0.23). One-way sensitivity analysis confirmed the stability of our results (Supplementary Figure 1B). Subgroup analyses stratified by various clinical variables indicated that patients benefit from high levels of intraepithelial CD3^+^ TILs with respect to regions (Europe; HR, 0.52; 95%CI, 0.46-0.58; P_HR_<0.001; I^2^=87.9%), methods of specimen processing (paraffin-embedded; HR, 0.30; 95%CI, 0.05-0.44; P_HR_=0.013; I^2^=93.0%), cut-off value (others; HR, 0.28; 95%CI, 0.14-0.55; P_HR_<0.001; I^2^=85.5%), number of patients (<150; HR, 0.42; 95%CI, 0.28-0.64; P_HR_<0.001; I^2^=99.4%), or histology (serous >66.7%; HR, 0.53; 95%CI, 0.47-0.59; P_HR_<0.001; I^2^=80.6%). The subgroup analyses were displayed in Figure 2C.

### CD8^+^ T lymphocytes

**Figure 3:**
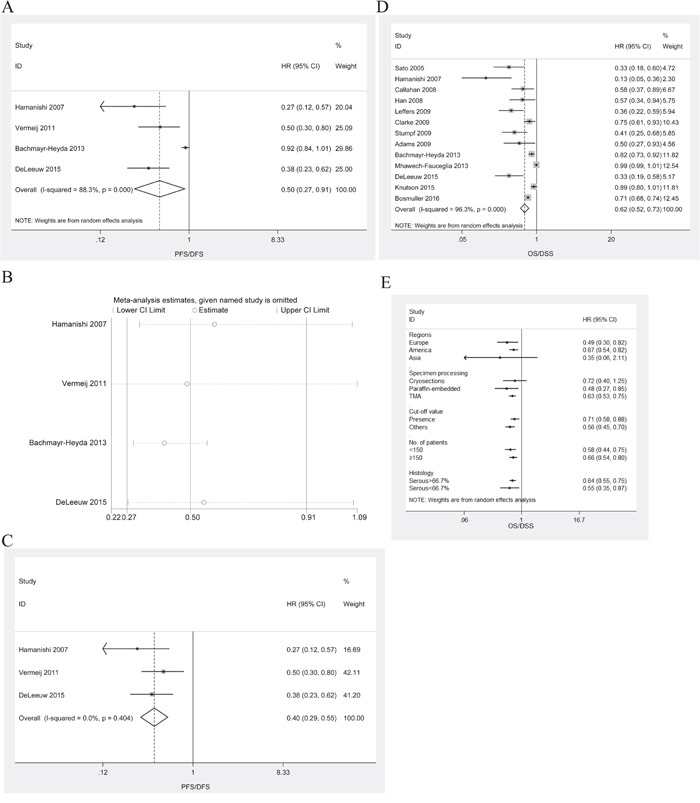
Meta-analysis of the HR for PFS/DFS and OS/DSS for ovarian cancer patients depending on intraepithelial CD8+ TILs status **A**. PFS/DFS for ovarian cancer patients, random effects model. **B**. Confirmation of the stability of the pooled results by one-way sensitivity analysis. **C**. PFS/DFS for ovarian cancer patients in the absence of the study by Mhawech-Fauceglia, fixed effects model. **D**. OS for ovarian cancer patients, random effects model. **E**. Subgroup analysis stratified by various clinical variables.

HRs for PFS/DFS were available in 4 studies [10, 18, 20, 22]. The estimated pooled HR for all studies suggested a significantly decreased risk of disease progression in patients with intraepithelial CD8^+^ TILs (Figure 3A; HR, 0.50; 95%CI, 0.27-0.91; P_HR_<0.022; I^2^=88.3%; random effects model). Publication bias existed (P_Begg_=0.308, P_Egger_=0.011). The trim-and-fill analysis indicated that there might be no missing studies (Supplementary Figure 2A). One-way sensitivity analysis indicated that the work by Mhawech-Fauceglia el al. had a significant influence on the estimated pooled HR for all studies (Figure 3B). In the absence of the study by Mhawech-Fauceglia, the estimated pooled HR indicated that the high intraepithelial CD8+ TILs were still associated with improved PFS/DFS in ovarian cancer patients (Figure 3C; HR, 0.40; 95%CI, 0.29-0.55; P_HR_=0.001; I^2^=0.0%; fixed effects model).

HRs for OS/DSS were available in 13 studies [8, 10, 11, 13–17, 19, 20, 22, 24, 25]. The estimated pooled HR for all studies suggested a significantly decreased risk of death in patients with intraepithelial CD3^+^ TILs (Figure 3D; HR, 0.62; 95%CI, 0.52-0.73; P_HR_<0.001; I^2^=96.3%; random effects model). Publication bias existed (P_Begg_=0.36, P_Egger_=0.007). The trim-and-fill analysis indicated that there might be no missing studies (Supplementary Figure 2B). One-way sensitivity analysis confirmed the stability of our results (Supplementary Figure 3). Subgroup analyses stratified by various clinical variables indicated that patients benefit from high levels of intraepithelial CD8^+^ TILs with respect to regions (HR, 0.49; 95%CI, 0.30-0.82; P_HR_=0.006; I^2^=83.0% for Europe; and HR, 0.67; 95%CI, 0.54-0.82; P_HR_<0.001; I^2^=86.6% for America), methods of specimen processing (HR, 0.48; 95%CI, 0.27-0.85; P_HR_=0.012; I^2^=89.8%, for paraffin-embedded; and HR, 0.63; 95%CI, 0.53-0.75; P_HR_<0.001; I^2^=80.2%, for TMA), cut-off value (HR, 0.71; 95%CI, 0.58-0.88; P_HR_=0.001; I^2^=87.8%, for presence; and HR, 0.56; 95%CI, 0.45-0.70; P_HR_=0.001; I^2^=81.7%, for others), number of patients (HR, 0.58; 95%CI, 0.44-0.75; P_HR_=0.001; I^2^=98.1%, for <150; and HR, 0.66; 95%CI, 0.54-0.80; P_HR_=0.001; I^2^=78.0%, for≥150), or histology (HR, 0.64; 95%CI, 0.55-0.75; P_HR_=0.001; I^2^=79.8%, for serous >66.7%; and HR, 0.55; 95%CI, 0.35-0.87 P_HR_=0.011; I^2^=92.3%, for serous<66.7%). The subgroup analyses were displayed in Figure 3E.

### FoxP3^+^ Treg lymphocytes

**Figure 4:**
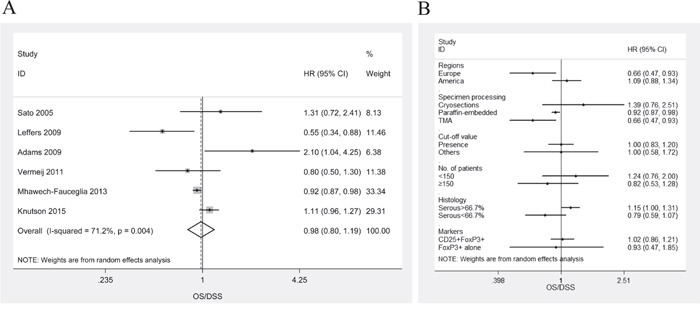
Meta-analysis of the HR for OS/DSS for ovarian cancer patients depending on FoxP3+ Treg TILs status **A**. OS/DSS for ovarian cancer patients, random effects model. **B**. Subgroup analysis stratified by various clinical variables.

HRs for OS/DSS were available in 6 studies [8, 14, 17–19, 24]. The estimated pooled HR for all studies suggested that the risk of death was not associated with intraepithelial FoxP3^+^ TILs in ovarian cancer patients (Figure 4A; HR, 0.98; 95%CI, 0.80-1.19; P_HR_=0.83; I^2^=71.2%; random effects model). There was no publication bias (P_Begg_=0.45, P_Egger_=0.66). One-way sensitivity analysis confirmed the stability of our results (Supplementary Figure 4). Subgroup analyses stratified by various clinical variables indicated that patients benefit from high levels of intraepithelial CD8^+^ TILs with respect to regions (Europe; HR, 0.66; 95%CI, 0.47-0.93; P_HR_=0.017; I^2^=15.8%), methods of specimen processing (HR, 0.92; 95%CI, 0.27-0.85; P_HR_=0.008; I^2^=23.2%, for paraffin-embedded; and HR, 0.66; 95%CI, 0.47-0.93; P_HR_=0.017; I^2^=15.8%, for TMA), or histology (serous >66.7%; HR, 1.15; 95%CI, 1.00-1.31; P_HR_=0.047; I^2^=38.1%). The subgroup analyses were displayed in Figure 4B.

### CD103^+^ T lymphocytes

**Figure 5:**
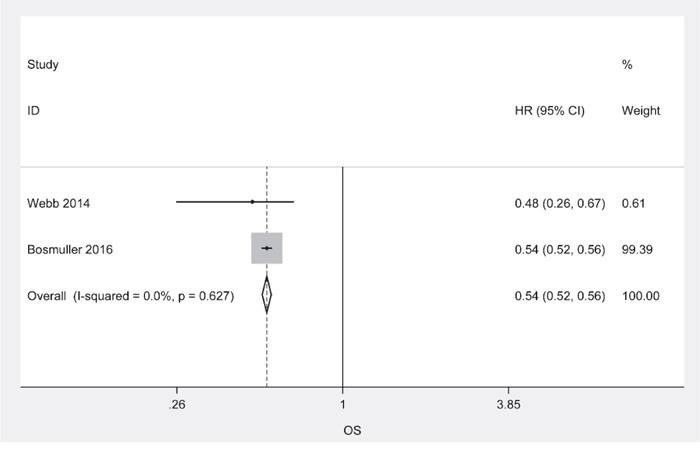
Meta-analysis of the HR for OS/DSS for ovarian cancer patients depending on CD103+ TILs status, random effects model

HRs for OS/DSS were available in 2 studies [21, 25]. The estimated pooled HR for these two studies suggested a significantly decreased risk of death in patients with intraepithelial CD103^+^ TILs (Figure 5; HR, 0.54; 95%CI, 0.52-0.56; P_HR_<0.001; I^2^=0.00%; fixed effects model).

### CD8^+^/FoxP3^+^ ratio

**Figure 6:**
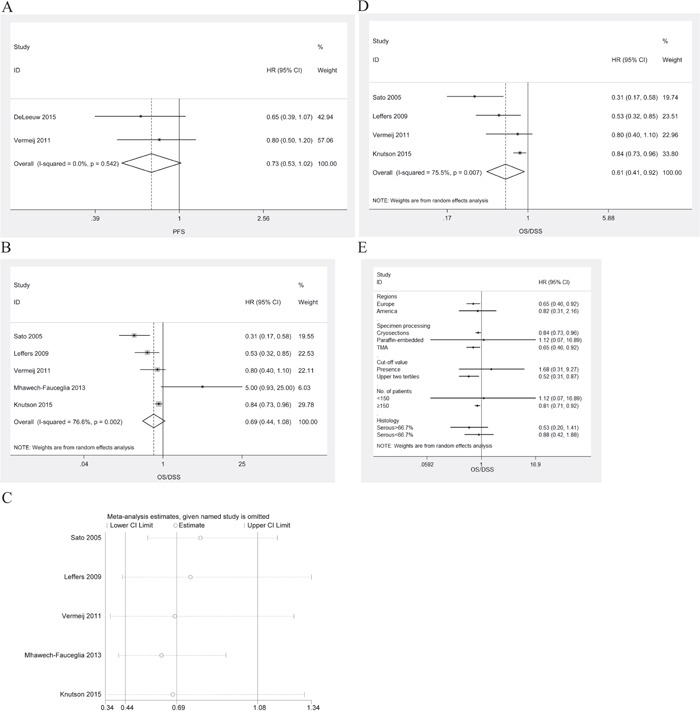
Meta-analysis of the HR for PFS/DFS and OS/DSS for ovarian cancer patients depending on CD8+/FoxP3+ ratio status **A**. PFS/DFS for ovarian cancer patients, fixed effects model. **B**. OS for ovarian cancer patients, random effects model. **C**. Confirmation of the stability of the pooled results by one-way sensitivity analysis. **D**. OS/DSS for ovarian cancer patients in the absence of the study by Mhawech-Fauceglia, random effects model. **E**. Subgroup analysis stratified by various clinical variables.

HRs of intraepithelial CD8^+^/FoxP3^+^ ratio for PFS/DFS were available in 2 studies [18, 22]. The estimated pooled HR for these two studies suggested that the risk of disease progression was not associated with intraepithelial CD8^+^/FoxP3^+^ ratio in ovarian cancer patients (Figure 6A; HR, 0.73; 95%CI, 0.53-1.02; P_HR_=0.064; I^2^=0.0%; fixed effects model).

HRs of intraepithelial CD8^+^/FoxP3^+^ ratio for OS/DSS were available in 5 studies [8, 14, 18, 19, 24]. The estimated pooled HR for all studies suggested that the risk of death was not associated with intraepithelial CD8^+^/FoxP3^+^ ratio in ovarian cancer patients (Figure 6B; HR, 0.69; 95%CI, 0.44-1.08; P_HR_=0.102; I^2^=76.6%; random effects model). There was no publication bias (P_Begg_=1.000, P_Egger_=0.708). One-way sensitivity analysis indicated that the work by Mhawech-Fauceglia el al. had a significant influence on the estimated pooled HR for all studies (Figure 6C). In the absence of the study by Mhawech-Fauceglia, the estimated pooled HR indicated that the high CD8^+^/FoxP3^+^ ratio was associated with improved OS/DSS in ovarian cancer patients (Figure 6D; HR, 0.61; 95%CI, 0.41-0.92; P_HR_=0.02; I^2^=75.5%; random effects model). Subgroup analyses stratified by various clinical variables indicated that patients benefited from high intraepithelial CD8^+^/FoxP3^+^ ratios with respect to regions (Europe; HR, 0.65; 95%CI, 0.46-0.92; P_HR_=0.015; I^2^=24.1%), methods of specimen processing (TMA; HR, 0.65; 95%CI, 0.46-0.92; P_HR_=0.015; I^2^=24.1%), cut-off value (upper two tertiles; HR, 0.52; 95%CI, 0.31-0.87; P_HR_=0.012; I^2^=63.4%), or number of patients (≥150; HR, 0.81; 95%CI, 0.71-0.92; P_HR_<0.001). The subgroup analyses were displayed in Figure 6E.

### CD8^+^/CD4^+^ ratio

HRs of intraepithelial CD8^+^/CD4^+^ ratio for OS were available in 2 studies [8, 24]. The estimated pooled HR for all studies suggested that the risk of death was not associated with intraepithelial CD8^+^/CD4^+^ ratio in ovarian cancer patients (Supplementary Figure 5; HR, 0.48; 95%CI, 0.21-1.11; P_HR_=0.086; I^2^=81.7%; random effects model).

### Global analysis of stromal CD3^+^ or CD8^+^ TIL effects on survival

HRs for PFS were available in 2 studies [8, 13]. The estimated pooled HR for all studies suggested that the risk of death was not associated with stromal CD3^+^ or CD8^+^ TILs in ovarian cancer patients (Supplementary Figure 6; HR, 0.82; 95%CI, 0.58-1.16; P_HR_=0.258; I^2^=24.8%; fixed effects model).

## DISCUSSION

The present meta-analysis is based on a large pool of clinical studies (2903patients) and differs from the previous meta-analysis in 2012 by Hwang et al. [28], which considered smaller series, and only assessed the prognostic value of CD3^+^ TILs and CD8^+^ TILs. Here, we identified 21 studies that evaluated the prognostic significance of different TIL subsets. It provides evidence that high densities of intraepithelial CD3^+^, CD8^+^, or CD103^+^ TILs alone are indicative of improved survival, but the presence of FoxP3^+^ TILs (Treg) alone, CD8^+^/FoxP3^+^ ratio, and CD8^+^/CD4^+^ ratio are not associated with the prognosis.

Since the landmark study by Zhang et al. [3], much work has been entered into the exploration of the prognostic significance of TILs in ovarian cancer. Collectively, the location, subtype, and density of TILs are major determinants of the prognostic value of TILs in ovarian cancer [3, 8–27]. Several studies have indicated that the exact location of TILs within tumor mass is critical for the prognostic effect in ovarian cancer [8, 13]. As evidenced by our meta-analysis, intraepithelial TILs other than stromal TILs are associated with favorable prognosis in ovarian cancer, emphasizing the importance of evaluating the location of TILs within the tumor microenvironment. Several attempts have been made to elucidate the mechanisms that promote the infiltration and localization of TILs to tumor islets in ovarian cancer. Callahan et al. [11] showed that tumor cell expression of HLA-DMB was associated with increased numbers of CD8^+^ TILs. Equivalently, by comparing gene expression profiles of 25 tumors containing low and 24 tumors containing high numbers of CD8+ TILs, Leffers et al. [29] identified 320 genes and 23 pathways differentially expressed which might contribute to or impede recruitment of lymphocytes into serous ovarian cancer. Additionally, Webb et al. [21] performed analysis on 497 EOC samples and found that CD103+ TILs were preferentially localized to epithelial regions of tumors. Despite these findings, the mechanisms that promote the infiltration and localization of TILs to tumor islets are still largely elusive.

In the present study, we evaluated the prognostic value of four subsets of TILs and our data indicated that intraepithelial CD3^+^, CD8^+^, or CD103^+^ TILs alone were indicative of improved survival, but the presence of FoxP3^+^ TILs (Treg) alone was not associated with the prognosis. Indeed, the prognostic value of intraepithelial Treg infiltration in ovarian cancer is still in debate [5, 30]. Some studies showed that Treg infiltration was associated with decreased overall survival in ovarian cancer [17, 30]. However, other studies failed to uncover such an association [8, 18, 24]. Moreover, a work by Leffers et al. [14] suggested a positive effect of Treg infiltration on the survival of ovarian cancer patients. These discrepancies may be partly attributed to differences on the regions of study population, the method for specimen processing, and the histology of ovarian cancer patients, as evidenced by our subgroup analysis. Furthermore, the difference on the selection of Treg markers in each study may also contribute to the contradictory findings. The reason is that the expression of FoxP3 by Treg cells may not be stable and that there is a great degree of flexibility in their differentiation options [31]. In addition, we also evaluated the prognostic value of ratios between different TIL subsets in ovarian cancer. Our results revealed that CD8^+^/FoxP3^+^ ratio and CD8^+^/CD4^+^ ratio were not associated with the prognosis of ovarian cancer. However, subgroup analysis suggested that patients benefited from high intraepithelial CD8^+^/FoxP3^+^ ratios with respect to regions (Europe), method of specimen regions (TMA), cut-off value (upper two tertiles), or number of patients (≥150). These discrepancies may be rooted in the variability in factors mentioned above.

Certain limitations must be considered when interpreting the pooled findings. First, the present meta-analysis is based on the data from studies whose results have been published, and the updated individual patient data were not available. Use of updated individual data may further improve the accuracy and reduce the uncertainty of the pooled findings. Second, significant heterogeneity existed in our study. Although meta-regression didn't identify any factors associated with HR estimates (data not show), variability in methods of specimen processing, cut-off value, selection of markers, sample size, histology of ovarian cancer, patient populations, and study design may give rise to the heterogeneity. Thus, large multicenter prospective studies based on homogeneous populations are needed to validate the prognostic value of TILs in ovarian cancer. Third, publication bias is another concern. We attempted to identify all relevant studies, but unavoidably, some studies could still be missing. Missing articles may contain negative results that could decrease the prognostic power of TILs. Additionally, other subsets of TILs with prognostic significance, for example PD-1^+^/PD-L1^+^ TILs [23, 27], are not included in our meta-analysis because of the limited number of published studies. Hopefully, the above findings could facilitate the international activity of “Immunoscore validation task force” in ovarian cancer [32].

In conclusion, TILs are of prognostic significance in ovarian cancer. The prognostic effects of TILs on ovarian cancer are dependent on the proportion of the different TIL subsets present instead of on the presence of a particular subset alone. In the future, the detection of different TIL subsets may serve as a tool to guide treatment in ovarian cancer patients [4, 5]. To achieve this goal, well-designed, randomized controlled trials in which therapy decision making is based on TILs status, are required to confirm current findings.

## MATERIALS AND METHODS

### Search strategy

A literature search (last search updated to Aug.16 2016) in Pubmed, ISI Web of Science, and Medline for articles addressing the prognostic significance of TILs in ovarian cancer was performed using the following keywords: (“tumor infiltrating lymphocytes” OR “T lymphocytes” OR “T cells” OR “regulatory T cells” OR “Treg”) AND (“ovarian cancer” OR “ovarian tumor” OR “ovarian carcinoma” OR “ovarian neoplasms”). Additionally, references lists of retrieved articles were checked for any possible eligible studies. The results were limited to peer-reviewed, English language reports.

### Eligibility criteria

The studies were deemed eligible if they reported survival data in ovarian cancer patients stratified by TIL status and provided sufficient data for determining an estimate of hazard ratio (HR) and a 95% confidence interval (CI). All studies were carefully reviewed to avoid inclusion of duplicate data. When the patient populations overlapped with patients in other included studies, only the most recent or most complete study was included to avoid duplications.

### Data extraction and outcomes

The data extracted for this meta-analysis included the author's names, year of publication, country and number of patients analyzed, tumor stage, grade, histology, method of specimen processing, cut-off value of TILs, phenotype and location of TILs. We also recorded progression free survival (PFS), disease free survival (DFS), overall survival (OS), disease specific survival (DSS), HR, and 95% CI if available.

### Statistical analysis

HR of each study was extracted either directly form the original report or calculated using the method proposed by Parmar and colleagues. The potential heterogeneity between studies was assessed by the Cochran's Q-test and expressed by the I^2^ index. The pooled HR for survival was calculated by fixed-effects model when the I^2^≤50%. Otherwise, random-effects model was used. Publication bias was assessed by the funnel plot and the Egger's and Begg's test. The impact of publication bias on the pooled HR was evaluated with the trim-and-fill method. Moreover, one-way sensitivity analysis was performed to assess the stability of the results. When the number of included studies was less than three, one-way sensitivity analysis was not performed. All statistical tests were conducted with STATA version 11.0.

## SUPPLEMENTARY MATERIALS FIGURES AND TABLES


