# Supplementary material for: The prognostic value of tumor-infiltrating T lymphocytes in ovarian cancer
Source: Oncotarget. 2017 Jan 31;8(9):15621–31. doi: 10.18632/oncotarget.14919 (PMC5362510; doi:10.18632/oncotarget.14919)
Supplement: Supplementary file 1 [file oncotarget-08-15621-s001.pdf]

The prognostic value of tumor-infiltrating T lymphocytes in ovarian cancer

SUPPLEMENTARY FIGURES

A

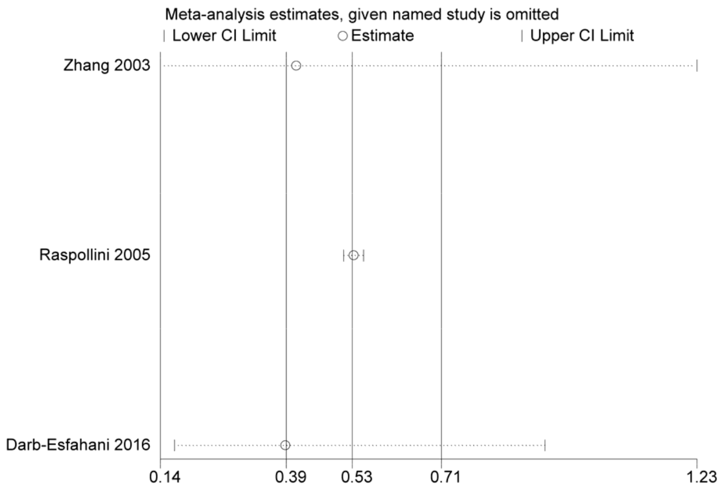

B

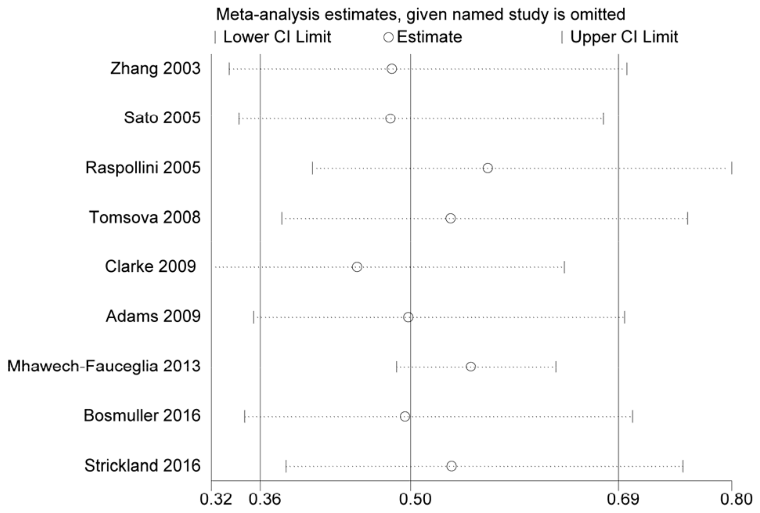

Supplementary Figure 1: One-way sensitivity analysis confirmed the effect of intraepithelial $CD3^{+}$ TILs on PFS **A.** and OS **B.** in ovarian cancer.

A

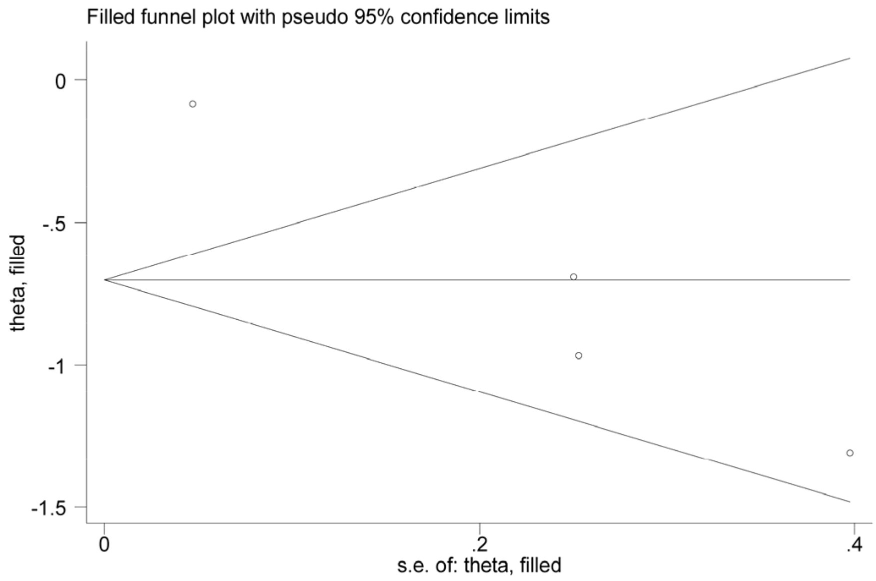

B

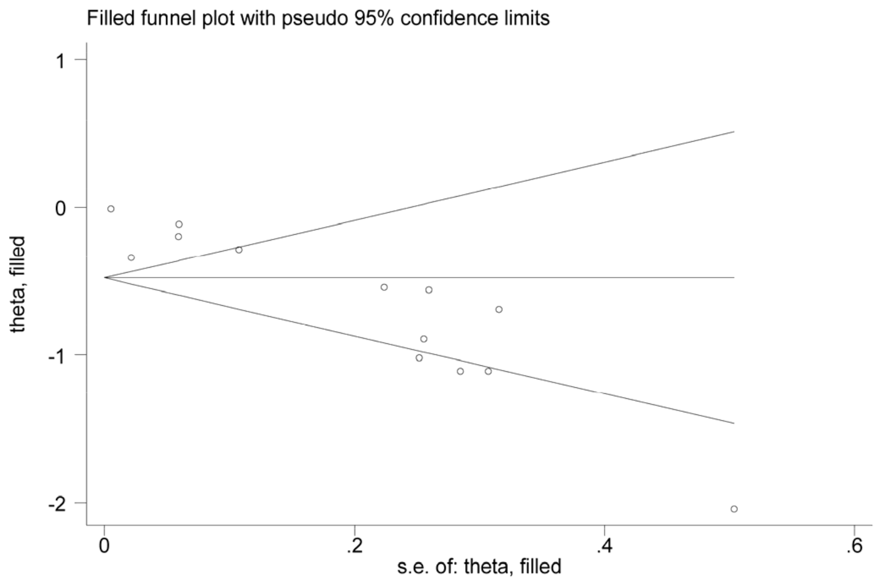

Supplementary Figure 2: The trim-and-fill analysis indicated that there might be no missing studies for both PFS **A.** and OS **B.**

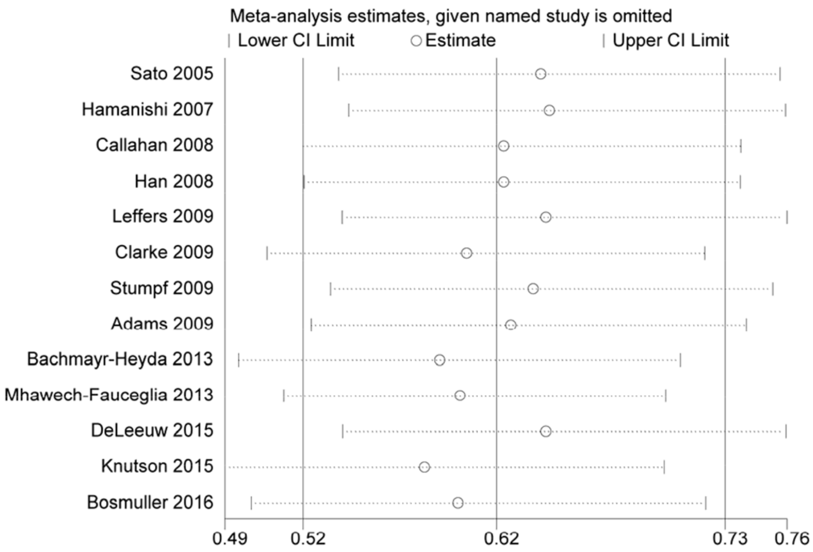

Supplementary Figure 3: One-way sensitivity analysis confirmed the effect of intraepithelial CD8+ TILs on OS in ovarian cancer.

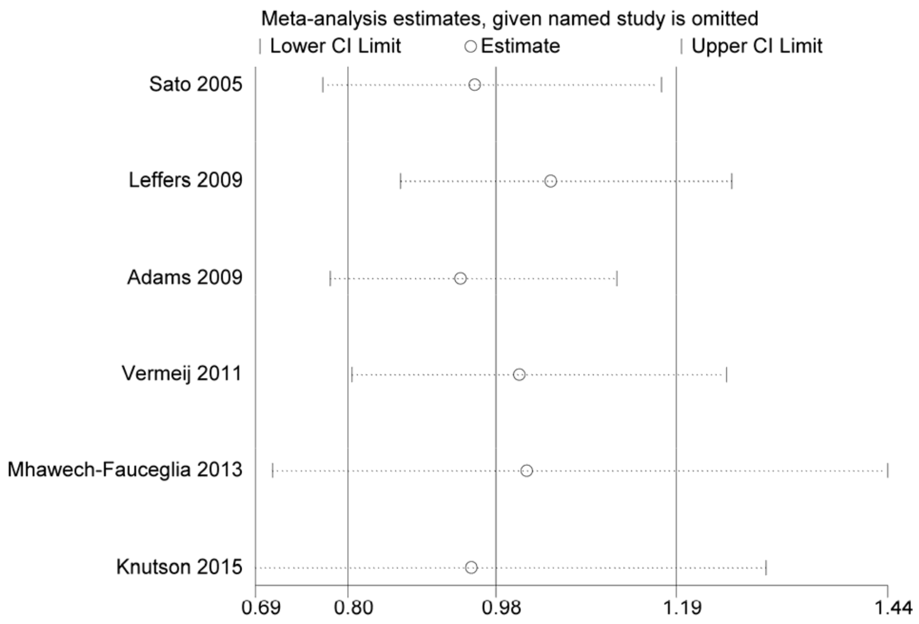

Supplementary Figure 4: One-way sensitivity analysis confirmed the effect of intraepithelial FoxP3^{+} TILs on OS in ovarian cancer.

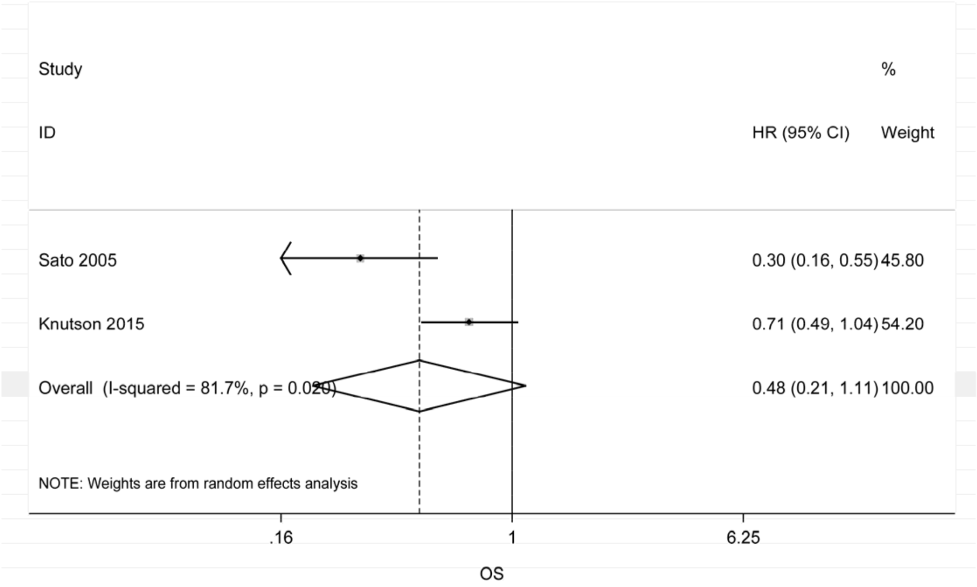

Supplementary Figure 5: Meta-analysis of the HR for OS/DSS for ovarian cancer patients depending on CD8^{+}/CD4^{+} ratio status, random effects model.

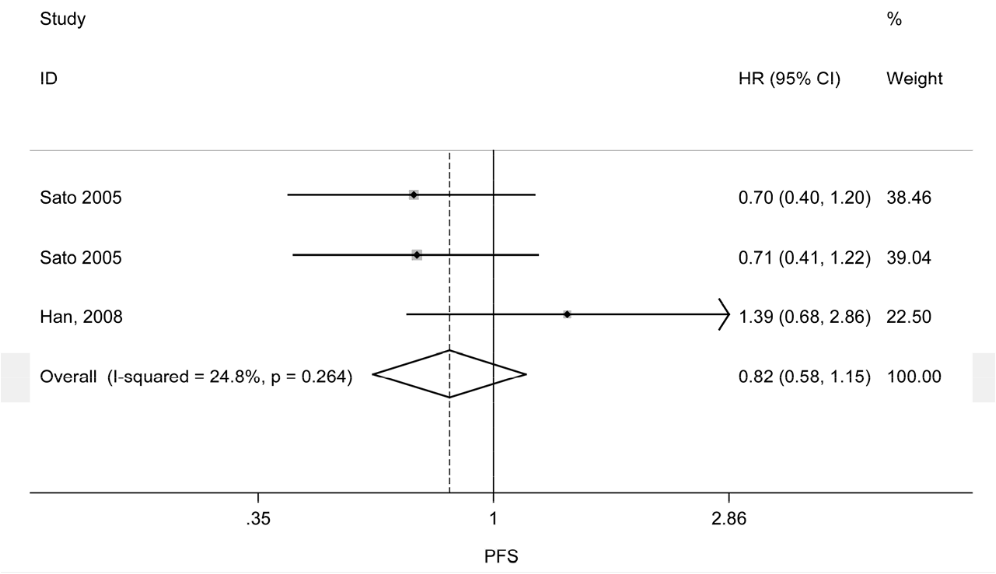

Supplementary Figure 6: Meta-analysis of the HR for OS/DSS for ovarian cancer patients depending on stromal TILs status, fixed effects model.
